# Floret-like multinucleated giant cells in neurofibroma

**DOI:** 10.1186/1746-1596-2-47

**Published:** 2007-12-08

**Authors:** Sameer Singh Shaktawat, Dariusz Golka

**Affiliations:** 1 Department of Histopathology, Central Manchester and Manchester Children's University Hospital NHS Trust, Manchester, UK; 2 Department of Histopathology, Blackpool Victoria Hospital, Blackpool, UK

## Abstract

This short report discusses a case of neurofibroma containing floret-like multinucleated giant cells. This being the second such case in the literature. Floret-like multinucleated giant cells have been reported in gynaecomastia and neurofibroma in neurofibromatosis type 1. These cells have been reported in uncommon soft tissue tumours including pleomorphic lipoma, giant cell collagenoma, giant cell fibroblastoma and giant cell angiofibroma. We recommend these cells to be interpreted carefully keeping in mind the rare malignant change in neurofibromas. Immunohistochemistry would help in defining the nature of such cells.

## Findings

**Figure 1:**
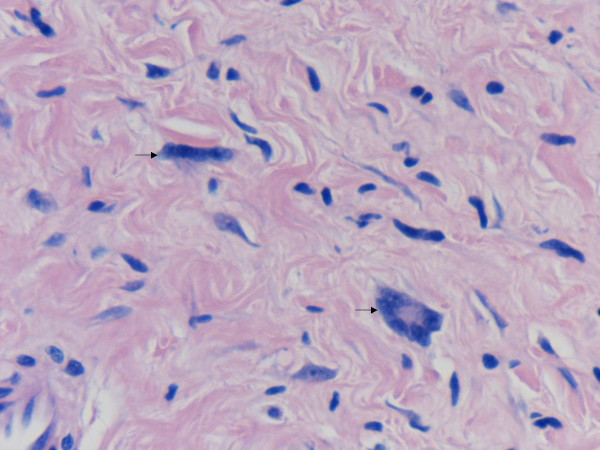
High magnification aspect of the neurofibroma showing floret-like multinucleated giant cells (arrows).

**Figure 2:**
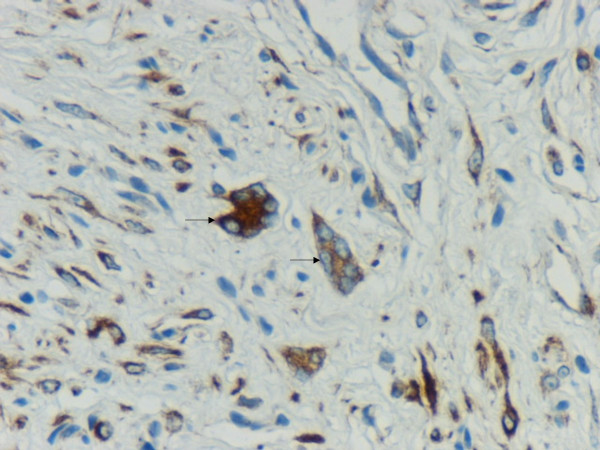
Vimentin positive floret-like multinucleated giant cells (arrows), in a background showing vimentin positive spindle cells.

**Figure 3:**
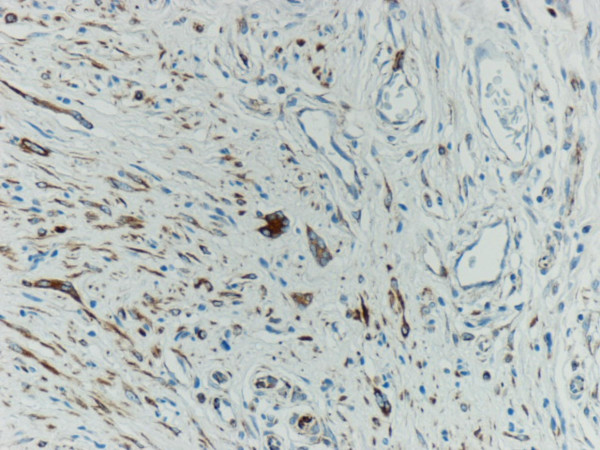
CD 34 positive cells and multinucleated giant cells in the neurofibroma.

We observed a case of cutaneous neurofibroma in a 73-year-old male from the upper back with a history of neurofibromatosis, showing numerous floret-like multinucleated giant cells (FMGCs). This intriguing but rare observation has been reported in neurofibroma only once before in a patient of type 1 neurofibromatosis (NF1) [1]. The tumour in our case was a 12 × 10 × 10 mm skin covered nodule, which on microscopy showed features of a diffuse neurofibroma and was present in the dermis and subcutaneous tissue. In low power view, the tumour was composed of spindle cells admixed with a few cells having hyperchromatic nuclei. The striking feature was the presence of numerous FMGCs with scant cytoplasm and peripherally arranged nuclei in the intervening stroma (Figure 1). These were positive with vimentin (Figure 2) and CD 34 (Figure 3) and negative with S-100 and CD 68. A subpopulation of non-Schwannian S-100 negative and CD 34 positive dendritic cells have been described in neurofibromas [2]. The spindle cells and the cells with hyperchromatic nuclei were positive with S-100, vimentin and CD 34 (Figure 3) and were negative with CD 68. Mitoses were absent and no nuclear atypia was recognised.

The presence of FMGCs in sporadic neurofibroma, a rare finding, adds to the growing list of soft tissue tumours with FMGCs, which include pleomorphic lipoma, giant cell collagenoma and giant cell fibroblastoma [3-5]. FMGCs have also been described in giant-cell-rich variant of solitary fibrous tumour also known as giant cell angiofibroma [6,7]. Many of these uncommon neoplasms may represent histologic spectrum of CD 34-positive dendritic interstitial cell neoplasms [7].

FMGCs have been described in gynaecomastia [8-10] and neurofibroma in NF1. We suggest that more studies are needed to understand the diagnostic utility of FMGCs in NF 1, whether it's an uncommon finding or typical for NF 1.

Benign nerve sheath tumours of soft tissue can adopt unusual morphologic appearances that may cause diagnostic difficulties, such as multinucleation, bizarre nuclei, intranuclear vacuoles, and other degenerative changes [11]. The presence of FMGCs should be interpreted with care, keeping in mind the presence of malignant transformation of neurofibromas in NF1. Ancillary studies like immunohistochemistry would help to define the nature of these cells. The absence of cytological features like pleomorphism and mitosis with increase in proliferation markers (MIB1) would help in ruling out malignancy and avoid misinterpretation of FMGCs in neurofibromas.

The FMGCs in our case were mesenchymal or fibroblastic in origin and this case may represent a histologic variant of neurofibroma or is part of the spectrum of the CD 34-positive dendritic interstitial cell tumours. Further studies are needed to clarify the nature of this entity.

## Competing interests

The author(s) declare that they have no competing interests.

## Authors' contributions

SSS reported the case with DG and did a thorough literature search, preparing the manuscript. DG edited the manuscript and made required changes. Both read and approved the final manuscript.
